# Treatment-refractory catatonia in an adolescent with a *de novo SIK1* candidate variant: a case report of a multidisciplinary diagnostic odyssey

**DOI:** 10.3389/fpsyt.2025.1657837

**Published:** 2025-09-17

**Authors:** Emmanuelle Hodara, Alexis Seegan, Jianling Ji, Alexander Van Speybroeck

**Affiliations:** ^1^ Department of Psychiatry, Keck School of Medicine of USC, Los Angeles, CA, United States; ^2^ Department of Pediatrics, Children’s Hospital of Los Angeles, Los Angeles, CA, United States; ^3^ Department of Psychiatry, University of California, Irvine School of Medicine, Irvine, CA, United States; ^4^ Center for Personalized Medicine, Department of Pathology and Laboratory Medicine, Children’s Hospital Los Angeles, Los Angeles, CA, United States; ^5^ Department of Pathology, Keck School of Medicine, University of Southern California, Los Angeles, CA, United States; ^6^ Department of Pediatrics, Keck School of Medicine of USC, Los Angeles, CA, United States

**Keywords:** catatonia, treatment-refractory, electroconvulsive therapy (ECT), genetic work-up, *SIK1* candidate variant, case report

## Abstract

We report the case of an adolescent patient who developed severe, treatment-refractory catatonia with progressive neurological decline over five years following a brief, self-limited acute psychotic episode following a prodromal period emerging at age 11 with disorganized behavior and restricted, stereotyped interests. Her atypical presentation marked by prolonged mutism, function loss, and retrograde amnesia, unresponsive to standard therapies, showed dramatic improvement with a combination of memantine, oxcarbazepine, and electroconvulsive therapy (ECT). Trio-clinical exome sequencing revealed a *de novo* likely pathogenic variant in the *SIK1* gene, highlighting the utility of genomic diagnostics in complex neuropsychiatric presentations and emphasizing the ongoing need for multidisciplinary collaboration and advocacy in patient care.

## Introduction

Catatonia is a neuropsychiatric disorder characterized by motor, behavioral and autonomic abnormalities, historically linked to mood and psychotic disorders but increasingly recognized across a wide spectrum of medical and neurological conditions. While well-described, the mechanisms underlying catatonia remain poorly understood. While often considered treatable with benzodiazepines, electroconvulsive therapy (ECT) and *N*-methyl-D-aspartate (NMDA) antagonists, catatonia remains underdiagnosed leading to life-threatening complications (1).

In pediatric populations, catatonia is rare and may follow a distinct clinical course. Presentations can include acrocyanosis, automatic compulsive movements, schizophasia, regression of previously acquired skills or communicative abilities and urinary incontinence (2). Prolonged and treatment-refractory catatonia has a strong association with schizophrenia, autism spectrum disorder and other neurodevelopmental disorders (3, 4). Importantly, genetic contributions appear especially relevant in youth. In the largest pediatric case series to date, Raffin et al. reported a high prevalence of underlying genetic conditions among children and adolescents with catatonia, underscoring the need to consider genetic testing early in complex or atypical presentations (5). Genetic underpinnings of catatonia are beginning to be explored, with emerging reports linking variants in synaptic signaling (particularly GABAergic transmission), mitochondrial function and neurodevelopmental pathways to catatonic presentations (6).

There is no universally accepted definition of chronic or prolonged catatonia. Most available data are limited to case reports, in which symptom duration have been greater than 30 days (7). Here, we describe an adolescent with profound and prolonged catatonia spanning months and progressive neurological decline, including prolonged mutism, functional regression, and retrograde amnesia, following a brief psychotic episode. The patient’s worsening presentation and diagnostic odyssey spanned five years before she showed significant improvement with a combination of memantine, oxcarbazepine and ECT. After extensive diagnostic workup, a *de novo* likely pathogenic variant in *SIK1* was identified through trio-based clinical exome sequencing. *SIK1* (Salt-Inducible Kinase 1) encodes a serine/threonine kinase critical in neuronal development including neurogenesis, dendritic growth and synaptic plasticity. Pathogenic variants in *SIK1* are linked to a rare autosomal dominant early infantile epileptic encephalopathy, marked by early-onset refractory seizures, severe developmental delay, hypotonia and intellectual disability, and early death (MIM:616341) (8). Later-onset cases show global impairment, absent speech, poor eye contact, inability to walk, behavioral manifestations, and feeding tube dependence (9). This case illustrates the importance of considering genetic contributions in refractory neuropsychiatric presentations and highlights the role of multidisciplinary, precision-guided approaches in care.

## Case presentation

### Etiological considerations and diagnostic trajectory

Taylor (name changed for privacy) is an 18-year-old female from a Latino immigrant family, raised in a stable two-parent household with steady employment. She lived with her parents and the family dog, while her two much older brothers had already moved out of the home. There was no known personal or family psychiatric history. Prior to 2019, Taylor was healthy, meeting developmental milestones, and thriving academically and socially—an honors student, avid soccer player, and horse enthusiast with a cheerful disposition. Over the subsequent five years, she underwent multiple medical and psychiatric evaluations in response to progressive behavioral and functional changes.

At age 13, Taylor was hospitalized at Institution 1 after two months of progressive disorganization, hallucinations, bizarre behaviors, insomnia, unsteady gait, and loss of independent functioning including feeding, toileting and bathing. The episode was preceded by prodromal symptoms suggestive of an emergent psychotic disorder, including social withdrawal, severe anxiety and academic decline, likely triggered by severe verbal bullying. Given her age and lack of personal or family psychiatric history, extensive workup was conducted to rule out medical causes, including autoimmune encephalitis.

Comprehensive medical workup (**Table 1**) – including imaging, electroencephalography (EEG), broad autoimmune, paraneoplastic, metabolic and infectious studies – was largely unremarkable. Ultimately, she was given the diagnoses of schizophrenia and catatonia and transferred to an inpatient psychiatric service where she was treated with risperidone and lorazepam. She improved and was able to regain continence, eat, dress and bathe by herself, and walk with a stable gait. However, she had no reciprocal speech and only participated when prompted. During this admission, she was also diagnosed with autism spectrum disorder based on collateral information from her older brother who reported long-standing limited reciprocal speech since early childhood and restricted stereotyped interest in horses and specific video games. Review of her personal journals, comprising primarily lists and rarely personal reflections, demonstrated increased disorganization since age 11, when her handwriting changed and drawings declined. Her mother challenged the diagnosis, attributing the social disconnect between Taylor and her brother to a large age gap, and noting that Taylor had never required school accommodations or regional center support. Her medications were discontinued by the family a month after hospitalization due to perceived symptom resolution, and Taylor was not followed by outpatient psychiatry. Taylor never returned to school or socialization. With the onset of the COVID-19 lockdown, she was homeschooled thereafter.

**Table 1:** Summary of extensive diagnostic evaluation and treatment attempts.

Date: Institution	Chief complaint and/or presentation on admission
Test/Treatment	Results/Response & diagnostic note
2020: Institution 1 (23 Day Admission)	*Acute psychosis, confusion, gait instability, urinary incontinence*
CBC w. Diff and CMP	Normal
Additional Blood Work	ANA: Negative TSH: 0.65; Free T4: 0.97; T3: 0.68 Anti-TPO: 1.0
CSF Analysis	CSF: Colorless, <1 RBC, 1 WBC 1, 92% Lymphocyte, 1% PMN, 7% Monocyte, Glc: 55, CSF Pre-Albumin 8.6 High, CSF Albumin 61.9 NMDA IgG <1:1 Meningoencephalitis PCR panel: Negative WNV Antibody: Negative Fungal/Gram Stain: Not enough CSF Mayo Panel: Autoimmune Encephalitis Negative
Additional Infectious Disease Work-Up	Serum Bartonella: Negative Serum Mycoplasma: IgM + **Negative Titers, IgG –** TB Skin test: Negative RPR: Negative
Urine Analysis and Culture Urine Porphyrins	UA: **Color Brown and slightly cloudy**; **Ketones 40** Ux: Negative Urine Porphyrin I: 8.8 Normal (ref: 3.6-21.1) Urine Porphyrin III: 2.1 Normal (ref:<5.6) **Coproporphyrins III: 98.6 High** (ref: 4.8-88.6) Total Porphyrins: 140.4 Normal (ref<150) * These results are unlikely suggestive of porphyria and may represent effect of variety of stressors (liver dysfunction, medications/toxins, starvation/catabolism).
Urine Toxicology	Positive for Benzodiazepines; consistent with prescription use Lead Level <1.0
MRI Brain w/and w/o contrast	Normal: No significant signal or structural abnormalities identified. Artifacts from patient’s braces.
Vascular Territory Map (VTM)	No diffusion abnormalities, no infarcts or perfusion defects
CT Chest Abdomen and Pelvis	To evaluate malignancy and paraneoplastic syndrome: 1. Tiny hypodense nodules in bilateral lobes of thyroid 2. No evidence of malignancy in chest, abdomen or pelvis.
Thyroid US and Needle Biopsy	Multiple cysts with eccentric echogenicity demonstrating comet tailed artifact suggest of colloid cyst. Pathology report consistent with colloid cyst.
Video EEG	No areas of focal slowing or epileptiform abnormalities.
Haloperidol 0.73mg + Lorazepam 2mg	For agitation on admission, discontinued during video EEG
Risperidone 1mg morning and 1.5mg nightly + Lorazepam 2mg	For psychosis and catatonia, led to improved ADLs with limited speech when prompted, no reciprocal speech. Medications were discontinued after a month.
2021-2023: Outpatient	Patient homeschooled; 2021: minor horse-back riding accident with no loss of consciousness precipitating progressive neurological decline
07/2022: Psychotherapy	3 visits, discontinued because patient was not participatory
08/2022: PCP	Insecticide Exposure Panel: Negative Fragile X: Negative
09/2022: Neurology	Referred to Institution 2 for Genetics
12/2022: Endocrinology	Pituitary MRI: micro pituitary tumor, subclinical
1/2023: Institution 2	Copper: 122 Ceruloplasmin: 22 Lead: <2 Homocysteine: 6.9 MMA: 0.11 B12: 991 Plasma acylcarnitine: Normal Free and total carnitine: Normal Amino acids: Essentially normal for age
2/2023: Institution 2	Invitae Metabolic Panel: non-contributory with incidental findings > *FECH* Low penetrance Pathogenic Mutation associated with AR erythropoietic protoporphyria > *GALC* Benign Mutation Quest Urine Organic Acid: **Patterns of elevated organic acid does not suggest a specific inherited metabolic disorder** aconitic acid 79 (ref 13-72), adipic acid 10 (ref 0-9), 2-OH-glutaric: 24 (ref 1-14)
2023: PCP	Initiated Fluoxetine 10mg and increased to 20mg, later discontinued due to rash Initiated Sertraline 25mg twice daily, but discontinued because of dizziness ANA: Positive 1:40, Speckled ***Diagnosis of selective mutism and anxiety***
09/2023: Neurology	24hr EEG: **rare spike wave discharges maximal at C4/T4 observed primarily during brief periods of wakefulness out of sleep; suggestive of focal cerebral dysfunction and predisposition to focal onset seizures**
09/2023: Occupational Therapy	Demonstrates difficulty and detachment from all active participation in self-care areas: communication, eating, dressing, grooming, toileting, play behaviors, organization
2023: Neurofeedback (40 Sessions)	Early gains with increased attentiveness and self-initiating movements, following commands, periodic attempts at speaking with incoherent sentences, but eventual plateau ***Diagnosis of selective mutism, apraxia***
03/2024: Institution 3 (11 day Admission)	Complete Loss of Speech; New abnormal movements (rapid eye movements, tremors)
MRI Brain	No acute hemorrhage. No herniation. Brain is normal in signal intensity and morphology for age. No evidence of encephalitis.
EEG (30min, 24hr)	No interhemispheric asymmetries, areas of focal slowing, epileptiform discharges or seizures. Events of interest captured for leg tremor and eyelid drooping do not have epileptiform correlate.
Lab work	BMP Normal Testosterone: 34; Hydroxyprogesterone-17: 31 Estradiol: 44; FSH: 3.68; Prolactin: 15.9; LH: 4.8 TSH 1.09; Free T4: 16
Lorazepam	For catatonia, gradually up titrated to 5mq q6H with improved symptoms including purposeful behavior and increased verbalization. Family self-titrated down lorazepam to 2mg q6H
2024: Institution 4 (67 day Admission)	Severe malnutrition (BMI 14.4), loss of 30lbs, incontinence, inability to eat, drink, speak/walk or tend to ADLs
Severe Malnutrition/Refeeding	Initiated IV hydration, NG tube placement, NG feeds, nutritional support including multivitamin, thiamine and cholecalciferol. Monitored for Refeeding Syndrome
IV Lorazepam Trial	Lorazepam 2mg q6H with improved oral intake and started walking with assistance
IV Lorazepam	Titrated to 40mg daily (10mg q6H) and later down titrated to 24mg (6mg q6H). Initial improvement and subsequent relapse.
Risperidone	Increased from 1.5mg daily to 1mg twice daily and switched to 2mg nightly. Decreased posturing, resistance and negativism; increased facial expression and smiling.
Amantadine	Started at 50mg twice daily and increased to 100mg morning and 200mg nightly. Improved movement, able to scroll/type on tablet; subsequent relapse.
Valproic Acid	Trialed valproic Acid via NGT 250mg nightly, discontinued due to increase in tremors in bilateral upper and lower extremities.
EEG (30min, 24hr)	Abnormal EEG due to diffuse excess fast activity likely secondary to benzodiazepines. No focal or epileptiform features.
MRI Brain	No acute infarct, hemorrhage, hydrocephalus or abnormal enhancement of the brain.
US Pelvic	2.7cm left ovarian cystic focus, likely cystic follicle
Lumbar Puncture: Performed twice due to inadequate samples leading to delay in results	CSF: Autoimmune Encephalitis Mayo Panel: Negative MS Panel: Negative Paraneoplastic/Encephalitis: Negative ANA 1:160, speckled
Other Lab Workup	TB: negative Varicella: Negative Hepatitis: Negative Immunological: IgA, IgG, IgM within normal limit, cytokines panel and T cell subsets within normal limit Rheum: ANA Negative Metabolic: Urine porphyria Negative, MMA level, Homocysteine level, folate and vitamin levels within normal limit
Methylprednisolone Pulse 1g x5 days	Empiric treatment; improvement movement, decreased stereotypy; subsequent relapse.
IVIG Trial 40g x 2 days	Decreased rigidity; discontinued following negative autoimmune panel.
Whole Exome Sequencing	SIK1 likely pathogenic mutation; resulted after discharge from Institution 5
2025: Institution 5 (63 day Admission)	Transferred from Institution 4 for ECT
Memantine 10mg twice daily Oxcarbazepine 300mg daily	Discontinued amantadine and trialed memantine at 10mg twice daily and oxcarbazepine 300mg daily Marked improvement including sustained return of speech and regain capacity to self-consent to ECT
ECT x12	Using SigmaStim Σigma device, we started with bitemporal placement with brief pulse width at 2x the seizure threshold as recommended (10), with settings: Pulse Width 1ms, Frequency 20Hz, Duration 5.8s, Current 800mA > Anesthesia medication was methohexital 100 mg IV for induction. Elected to use rocuronium 40 mg IV as the paralytic agent initially due to her prolonged immobilization and concern for hyperkalemia due to succinylcholine. Reversal of rocuronium was done with sugammadex 400 mg IV. Continued improvement in speech, ADLs, and restoration of consciousness; reported anterograde amnesia.
2025: Outpatient	Maintenance ECT and Outpatient Follow-up
ECT Maintenance:	Initially every 4weeks and increased to every 2 weeks due to breakthrough symptoms and decline before next remitment. Overall improved functional recovery
Memantine 10mg twice daily Oxcarbazepine 600mg	Regression immediately following discontinuation; resumed both medications and oxcarbazepine increased from 300 to 600mg daily with improvement in self-initiated movement.

Bold text refers to pertinent positive work-up.

Between ages 15 and 17, Taylor experienced progressive cognitive and functional decline following a minor horseback riding accident. She reported worsening concentration, reading comprehension and memory, with speech becoming minimal, but never exhibited mood lability or psychosis. Multiple outpatient evaluations (**Table 1**) including neuroimaging, EEGs, metabolic and genetic panels, endocrinology and rheumatology studies, and insecticide exposure, were unrevealing. One 24-hour EEG showed rare spike-wave discharges (maximal at C4/T4), suggesting focal cerebral dysfunction and predisposition to focal seizures. Her primary care physician made the diagnosis of selective mutism, recommended cognitive behavioral therapy (CBT) and referred Taylor to a selective mutism clinic. Psychotherapy attempts were discontinued because Taylor was non-participatory. By April 2023, she became fully mute and diagnosed with apraxia and selective mutism. During this period, Taylor was still walking and eating independently. She underwent 40 neurofeedback sessions with early gains in attentiveness and movement which plateaued. Aside from one 20-minute episode of regained speech, she remained mute and disengaged. At age 17 (March 2024, Institution 3), Taylor was hospitalized for worsening regression including poor oral intake, immobility, rigidity and absent speech, accompanied by abnormal eye movements concerning for seizures. MRI brain and prolonged video EEGs were normal. Catatonia was explicitly diagnosed for the first time. High dose lorazepam transiently restored speech, the first meaningful improvement in years. However, follow-up was disrupted by a clerical error and transition out of pediatric services. Efforts to identify outpatient psychiatrists were unsuccessful reflecting the challenges posed by her atypical presentation and ongoing high-dose benzodiazepine regimen. With limited oversight, the family tapered Taylor’s dose due to sedation worsening her condition.

In October 2024, at age 18, Taylor was admitted to Institution 4 with severe malnutrition (BMI 14.4) following six months of decline marked by inability to eat, drink, or take medications, 30 lb weight loss, complete functional dependence and incontinence. On admission to the hospital, Taylor was completely disengaged, with minimal movements, responding to painful stimuli with mild groans. Her presentation with immobility, staring, posturing of wrists and hands, grimacing, rigidity, waxy flexibility, absence of speech and withdrawal was consistent with severe catatonia of unknown etiology. Given her recent evaluation at Institution 3, further testing was initially deferred; however, her fragmented care history and protracted clinical course including regression and loss of skills less consistent with an underlying psychiatric etiology for her catatonia prompted further workup (**Table 1**), including imaging, EEG, lumbar puncture, infectious and rheumatologic studies, and a trio-clinical exome sequencing. Repeat EEGs showed generalized fast activity (likely benzodiazepine-related), and MRI remained normal. Sequencing was prompted in part by a published report of an adolescent with a very similar presentation of treatment-refractory catatonia discovered to have a 22q11 deletion on whole exome sequencing (11). Clinical exome sequencing, completed post-discharge, revealed a *de novo* heterozygous variant in the *SIK1* gene prompting further investigation into its clinical significance, and possibly reframing her condition as a neurodevelopmental disorder with catatonic features.

### Treatment strategies and clinical course

On admission to Institution 4, Taylor required supportive care for her severe decompensation, including initiation of nasogastric feeds, intravenous fluids, and vitamin supplementation with monitoring for refeeding syndrome. Due to poor oral intake, Taylor had been off her medications, including benzodiazepines for almost two weeks by the time of her hospitalization. Lorazepam was initiated at 2mg IV and titrated to 40 mg daily in an effort to treat her catatonia. This resulted in a brief improvement with her Bush Francis Catatonia Rating Scale (BFCRS) (12) improved from 19 to 14 within days; however, she soon regressed, with reduced oral intake and increased rigidity, and BFCRS rising to 18.

While awaiting results of CSF autoimmune panel and genetic testing, empiric treatment for autoimmune encephalitis was initiated. A five day course of high dose methylprednisolone, followed by intravenous immunoglobulin led to modest but transient functional gains, including improved alertness and movement. Treatment was discontinued when encephalitis panel returned negative.

Additional psychotropic trials were attempted with limited benefit. Risperidone was titrated to 2mg nightly and well-tolerated, but produced little improvement. Valproic acid at 250mg nightly was discontinued after 3 days due to tremors. Amantadine started at 50mg twice daily and increased to 100mg morning and 200mg nightly led to brief improvement in alertness, initiation of purposeful movement, with improved BFCRS of 12 before rebounding to 18.

Given limited response to multiple neurotropic and immunologic agents and the recognition of ECT as the gold standard for treatment-resistant catatonia, Taylor was transferred to Institution 5 for ECT and continued feeding tube management. There, lorazepam was decreased to 24mg daily, and amantadine and risperidone were discontinued. Based on a published case of treatment-refractory catatonia, Taylor was started on memantine 10mg twice daily, and oxcarbazepine 300mg daily, later increased to 600mg daily (13). This combination produced her most sustained improvement, with restoration of spontaneous speech, improved and sufficient decisional capacity to consent to ECT. She subsequently underwent 12 sessions of bitemporal ECT, resulting in marked recovery described as “an awakening” (14). At this point, she reported anterograde amnesia spanning approximately three years, attributed to catatonia rather than ECT. Functionally, she regained independence in daily activities, expressed motivation to complete high school, and returned to her cheerful baseline.

Maintenance ECT was initially administered at four-week intervals, but breakthrough symptoms, including slowed processing, speech latency, regression in activities of daily living (ADLs), and recurrence of stereotypies emerged in the two weeks preceding next treatment. These symptoms also recurred immediately after memantine and oxcarbazepine were abruptly discontinued by an outpatient psychiatrist. Her current regimen presently consists of biweekly maintenance ECT alongside memantine, oxcarbazepine and lorazepam, with efforts underway to optimize neuromodulating medications and gradually taper lorazepam. This approach supports stable, though incomplete recovery.

## Discussion

This case describes an adolescent who developed severe, treatment-refractory catatonia with progressive neurological decline over five years following a brief, acute psychotic episode following a prodromal period with disorganized behavior and restricted, stereotyped interests. Her atypical presentation marked by prolonged mutism, function loss, and retrograde amnesia, unresponsive to standard therapies, showed dramatic but incomplete improvement with a combination of memantine, oxcarbazepine, and electroconvulsive therapy (ECT).

Over the years, Taylor received a wide range of diagnoses — many likely inaccurate — including schizophrenia, autism spectrum disorder, traumatic brain injury, selective mutism and catatonia, with only minimal improvement. The chronicity and refractoriness of her catatonic presentation prompted consideration of a broad differential diagnosis encompassing but not limited to primary psychiatric illness, especially since up to 20% of pediatric catatonia cases are secondary to medical conditions (15) and often have specific treatments (16, 17). Medical etiologies for her catatonia including structural abnormalities, epileptic, autoimmune, infectious, metabolic and toxicological causes were extensively worked-up, with no diagnostic evidence other than transient and partial recovery with immunologic therapies and antiepileptic agents. Psychiatrically, her course raised consideration of schizophrenia and autism spectrum disorder, but also less common syndromes such as pervasive refusal syndrome (PRS) and neurodevelopmental disorder with regression and abnormal movements, loss of speech and seizures (NEDAMSS). Pervasive refusal syndrome has been described in youth with severe functional decline, the core features of refusal across multiple domains (eating, mobilizing, communicating) typically emerge in the context of psychological stressors (commonly reported among asylum seekers) and without the neurological signs or fluctuating response to pharmacologic and neuromodulating interventions observed here (18). However, given the overlapping features of mutism, immobility, and withdrawal, some experts argue that PRS is better understood as a misnomer for catatonia rather than a distinct syndrome (18). Similarly, NEDAMSS, a recently described genetic disorder linked to *IRF2BPL* variants and characterized by developmental regression, movement abnormalities, and profound speech loss with phenotypic overlap with our patient’s course but different genetic variants (19). Taken together, these comparisons underscore the diagnostic complexity and highlight the need for careful longitudinal assessment before attributing catatonia solely to a primary psychiatric disorder, as well as consideration of neurodevelopmental and genetic etiologies, particularly given the high rates of pathogenic variants reported in pediatric catatonia (5).

Based on recent clinical exome sequencing, our current understanding is that her catatonia is secondary to a late-onset autism spectrum disorder, likely associated with a pathogenic variant in *SIK1*, potentially explaining her incomplete and transient response to multiple treatment regimens. Notably, Taylor’s most sustained improvement followed treatment with oxcarbazepine, raising the possibility that subclinical epileptiform activity underlies aspects of her catatonia. Taylor’s variant (NM 173354.5:c.1760G>A, p.Arg587Gln) has not been previously reported in affected individuals. It is absent in large germline population studies (gnomAD v4.1.0), though sequencing coverage at that position is limited. The substitution occurs at a moderately conserved position, and computational analyses suggest it may impact protein function. With both parents showing normal sequences at this locus, it is considered a *de novo* candidate variant to contribute to her presentation.

Pathogenic SIK1 variants are most often described in early-onset epileptic encephalopathy, though later-onset cases with broader neurodevelopmental impairment and behavioral features are also briefly mentioned in the literature but remain poorly characterized (8, 9). Using GeneMatcher (20), an online platform that connects clinicians and researchers sharing rare genetic variants, we identified nine global cases with *SIK1* pathogenic variants presenting atypically; none matched Taylor’s exact variant or symptoms. Across these reports, phenotypes were heterogeneous but generally included neurodevelopmental delay, seizures or abnormal EEG findings, and motor and speech impairment. One patient had autism and “staring spells” with abnormal but non-epileptic EEG, and another had speech delay, mild intellectual disability, and facial dysmorphisms. To our knowledge, catatonia has not previously been associated with SIK1 variants, suggesting that Taylor’s presentation may expand the recognized phenotypic spectrum. Nonetheless, causality cannot be inferred from a single case.

Beyond being a diagnostic odyssey with hope for a meaningful recovery, Taylor’s case highlights the clinical utility of genomic diagnostics in psychiatric diagnosis and treatment, and underscores the need for sustained, multidisciplinary advocacy in complex neuropsychiatric care.

Genetic findings may contribute important insights in diagnostically complex psychiatric presentations by clarifying underlying etiologies. Though the mechanistic pathways remain unclear, this case highlights the value of integrating early genetic testing into diagnostic workup for atypical first break psychosis, particularly when catatonic features are present. In youth with neurodevelopmental disorders, catatonia is often atypical and refractory to benzodiazepines, with emerging evidence supporting the efficacy of ECT in such cases (3). For Taylor, identification of a candidate variant in *SIK1* suggests a previously unrecognized neurodevelopmental etiology, suggesting that earlier trials of antiepileptic, glutamatergic agents, or ECT may have been beneficial. This is especially relevant in pediatric psychiatry, where atypical presentations often defy conventional categories. As exome sequencing becomes more accessible, it may help clarify etiology, inform treatment selection, and challenge the divide between “functional” and “organic” disorders, ushering in a more personalized era of psychiatric care.

Perhaps most critically, this case illustrates the consequences of fragmented care and premature diagnostic closure. Across top institutions, opportunities for coordinated, multidisciplinary oversight were repeatedly missed. Neurology teams repeatedly disengaged after ruling out primary structural or autoimmune disease. Outpatient psychiatrists were uncomfortable managing her case and medications. One provider, assuming her catatonia was secondary to schizophrenia, discontinued medications that had supported her functional recovery. Despite Herculean persistence from her family, it took years to reach a working diagnosis and initiate effective treatment. Taylor’s case is a stark reminder of the need for multidisciplinary persistence – bridging psychiatry, neurology and genetics– in complex cases where siloed care delays diagnosis, worsens symptoms, and leads to preventable decline.

## Patient perspective

Taylor and her family are encouraged by the meaningful progress she has made in recent months through a combination of pharmacotherapy and ECT. As described in the case, Taylor had been in a state of stupor for over three years, with no recollection of events since her steep cognitive decline began in 2021. When she emerged from this state at the age of 18, Taylor believes she was still 15. Since then, she has expressed renewed self-direction including a desire to finish high school and go to college. She enjoys activities including shopping, listening to music and singing. She has regained her happy disposition. However, it is apparent that her cognitive and motor functions are not at baseline: her processing and responses delayed, her actions slowed, her posture stooped with a hunched back and forward head, and her gait shuffling. These symptoms, along with intermittent stereotypies, become more pronounced in the week leading to her next ECT treatment, and remain present even on her best days.

The family has been very intrigued by the genetic findings discussed in this report, and wonders whether seizures, even undetected in prior video EEGs, may contribute to her condition. Their journey continues with regular maintenance ECT, continued adjustment of psychopharmacotherapies, and multidisciplinary support including physical, occupational and speech therapy.

## Conclusion

This case illustrates the complexity of pediatric catatonia, a syndrome increasingly recognized as arising from diverse medical and genetic etiologies rather than solely primary psychiatric disorders. In this context, we identified a *de novo* likely pathogenic SIK1 variant that may contribute to the patient’s phenotype and represents, to our knowledge, the first reported expansion of the SIK1 spectrum to include catatonia. The case also underscores the importance of sustained multidisciplinary follow-up across psychiatry, neurology, and genetics in complex diagnostic presentations to optimize outcomes and guide management.

## Data Availability

The original contributions presented in the study are included in the article/**Supplementary Material**. Further inquiries can be directed to the corresponding author.
